# Enhanced serum-based seed amplification assay for detecting propagative α-synuclein seeds in Parkinson’s disease

**DOI:** 10.1186/s40035-025-00488-3

**Published:** 2025-05-22

**Authors:** Yaoyun Kuang, Hengxu Mao, Wei Dai, Tingting Gan, Hao Lin, Jin Li, Xinling Yang, Pingyi Xu

**Affiliations:** 1 Department of Neurology, The First Affiliated Hospital of Guangzhou Medical University, Guangzhou, 510120 China; 2 Graduate School, Xinjiang Medical University, Urumqi, 830054 China; 3 The Second Affiliated Hospital of Xinjiang Medical University, Urumqi, 830054 China; 4 Department of Oncology, The Key Laboratory of Advanced Interdisciplinary Studies, The State Key Laboratory of Respiratory Disease, The First Affiliated Hospital of Guangzhou Medical University, Guangzhou, 510120 China

## Main text

Fibrillar aggregates of α-synuclein (αSyn) play a key role in the pathogenesis of Parkinson’s disease (PD) and other synucleinopathies, making them critical targets for diagnostic approaches. In this context, seed amplification assays (SAAs) have emerged as a promising diagnostic technique by exploiting the self-propagating nature of misfolded αSyn aggregates. SAAs have been successfully used to detect αSyn in various biological samples, including cerebrospinal fluid (CSF), skin, and blood [1]. Among these, blood-based diagnostics are particularly suitable for early detection and disease monitoring due to their non-invasive nature. However, the presence of numerous components, such as albumin and other inhibitory proteins, complicates the implementation of SAA in blood samples [2]. These components may influence the sensitivity by interfering with the aggregation of αSyn, which results in compromised detection efficiency.

To address this challenge, several strategies have been developed to overcome these inhibitors. For instance, the Okuzumi group employed immunoaffinity capture methods to isolate αSyn seeds from serum, while other studies successfully isolated neuronal-derived extracellular vesicles, effectively overcoming the inhibitory effects of serum components [3, 4]. Despite these advancements, the exact nature of the inhibitory substances in blood remains largely uncharacterized.

Given the biological relationship between blood αSyn and the pathogenesis of PD, we conducted an in-depth investigation into the inhibitory effects of human serum on αSyn aggregation. Building on the fractionation procedure described by Bellomo et al. and the role of lipoproteins in inhibiting αSyn aggregation, we first fractionated human serum from healthy controls (HCs) using centrifugal molecular weight cut-off (MWCO) filters [5]. This process generated five distinct fractions: whole serum, > 100 kDa, 30–100 kDa, 3–30 kDa, and < 3 kDa (Fig. S1a). After fractionation, we spiked serum retentates and filtrates into αSyn SAA reactions without seeds to assess their potential role in modulating the self-aggregation process, independent of αSyn seeds. Under these experimental conditions, whole serum and the > 100 kDa fraction significantly inhibited αSyn aggregation, whereas the smaller fractions exhibited aggregation levels similar to the PBS control (Fig. S1b). These results indicate that the > 100 kDa fraction plays a key role in preventing αSyn aggregation. To understand its composition, we used liquid chromatography-mass spectrometry analysis and identified over 250 proteins in this fraction (Table S1). The most abundant serum proteins were selected for further study, including apolipoproteins, α2-macroglobulin, and serum-purified transferrin (Fig. S1c) (full methodology in Materials and methods).

We first examined high-density lipoprotein (HDL) and low-density lipoprotein (LDL), which are the primary lipoproteins in human serum, along with their associated apolipoproteins. When 10 fg of human αSyn preformed fibrils (hPFFs) were added to each well (hPFFs control group), a sigmoidal increase in Thioflavin T (ThT) fluorescence was observed, indicating αSyn aggregation. In contrast, HDL and LDL both showed dose-dependent inhibition of αSyn aggregation. Specifically, HDL and LDL at 200 nmol/L induced a modest decrease in the protein aggregation rate (PAR) compared to the hPFFs control group, while aggregation was almost fully inhibited at 1 μmol/L. Meanwhile, the addition of PBS instead of hPFFs displayed no significant changes in fluorescence (Fig. S2a, c). In contrast, PAR analysis for transferrin and α2-macroglobulin at 200 nmol/L, 500 nmol/L, and 1 μmol/L showed no significant inhibition of αSyn aggregation compared to the hPFFs control group (Fig. S2e). To further explore the role of specific proteins in αSyn aggregation, ApoA1 and ApoE were immunoprecipitated from HC serum and the serum sample was subsequently incubated with hPFFs at 37 °C for 1 h. The depletion of ApoA1 and ApoE significantly accelerated αSyn aggregation, as evidenced by an increased PAR compared to groups of immunoprecipitation without antibodies (IP no Ab) and untreated serum control (Fig. S2f).

**Fig. 1:**
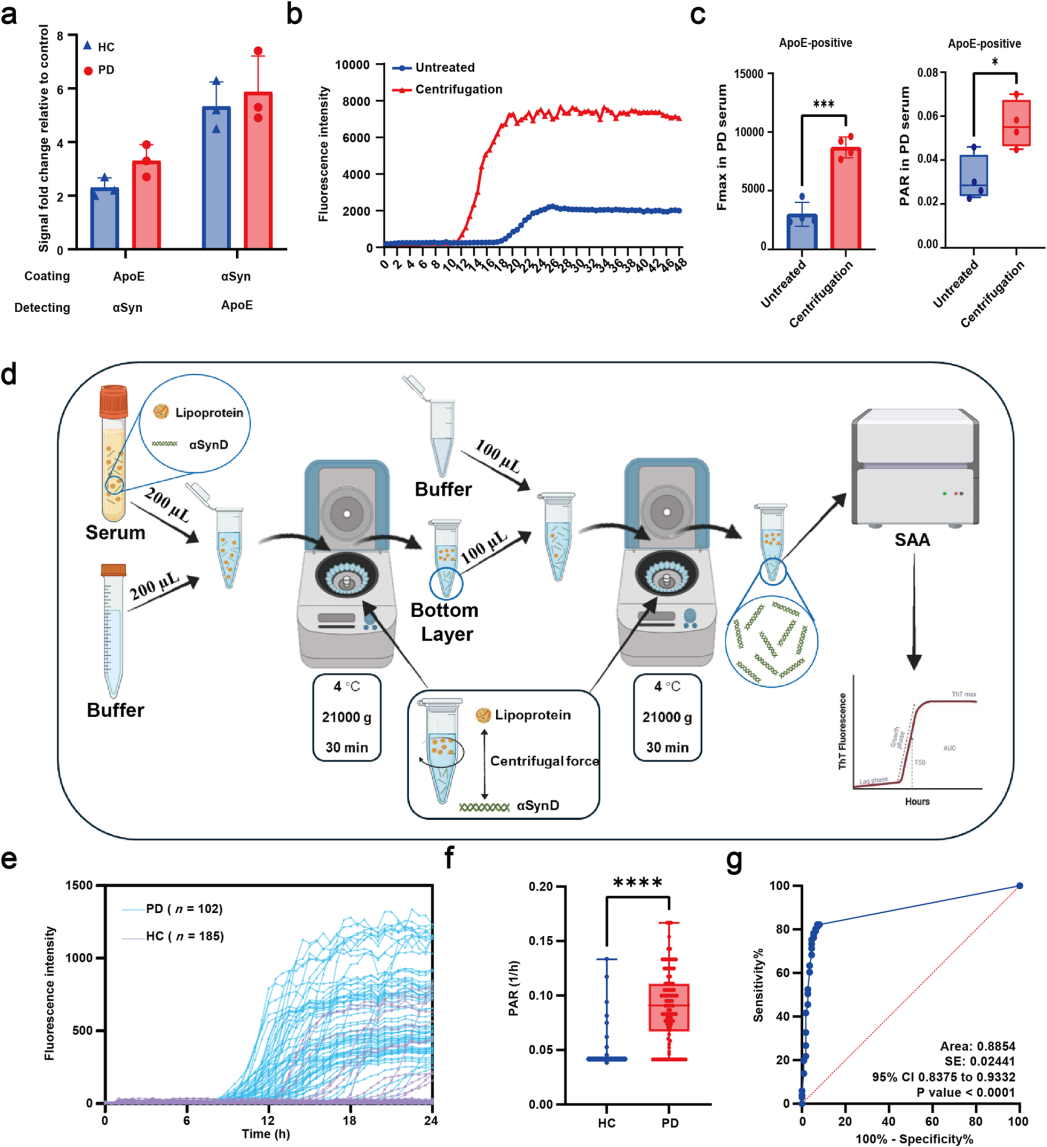
Detection of αSyn seeds in serum samples from PD patients using lipo-free SAA. **a** Detection of ApoE-αSyn interaction in HC and PD serum samples using ELISA. Left: αSyn detection on anti-ApoE-bound complexes. Right: ApoE detection on anti-αSyn-bound complexes (*n* = 3). **b** SAA kinetics in ApoE-positive vesicles from PD serum with or without centrifugation. **c** Comparison of *F*_max_ and PAR in ApoE-positive vesicles from PD serum after centrifugation (*n* = 4). **d** Schematic of serum preparation for lipo-free SAA. **e** SAA amplified αSyn seeds in lipo-free serum from 102 PD patients and 185 controls. **f** PAR comparison between PD and controls after lipo-free SAA. **g** ROC curve with AUC. Two-tailed Student’s *t*-test used. Data are presented as mean ± SD; *P < 0.05, **P < 0.01, ***P < 0.001. AUC, area under the curve; ApoE, apolipoprotein E; αSyn, alpha-synuclein; *F*_max_, maximum fluorescence intensity; HC, healthy control; PAR, protein aggregation rate; PD, Parkinson’s disease; ROC, receiver operating characteristic; SAA, seed amplification assay

A key question is why apolipoproteins affect αSyn seeding activity. αSyn colocalizes with apolipoproteins in human CSF, which may explain their inhibitory effects [5]. To investigate a similar interaction in serum, we used a two-step ELISA to examine the interaction between ApoE and αSyn in serum samples from PD patients and HCs. ApoE, which is partially produced by astrocytes and microglia, is known to directly bind to αSyn and regulate its aggregation [6]. In the first setup, ELISA plates were coated with an anti-αSyn antibody raised against total synuclein, and then serum samples were added. An anti-ApoE antibody was added to detect ApoE binding. In the second setup, plates were coated with anti-ApoE antibody and an anti-αSyn antibody was added to detect αSyn binding. Both setups showed clear ELISA signals in PD and HC serum samples, indicating a strong interaction between ApoE and αSyn (Fig. 1a). Interestingly, previous research has indicated that αSyn colocalizes with apolipoproteins in human CSF, and that centrifugation can potentially reverse the inhibitory effects of apolipoproteins on αSyn seeding by detaching them from αSyn aggregates [6]. To investigate this further, we performed IP on PD serum to isolate ApoE-positive vesicles. Subsequently, these vesicles were subjected to high-speed centrifugation for 15 min. We then assessed the αSyn seeding activity in the resulting bottom supernatant using SAA. The results showed that centrifugation significantly enhanced αSyn seeding activity, as evidenced by increased maximum fluorescence intensity (*F*_max_) and PAR compared to uncentrifuged controls (Fig. 1b, c). These findings suggest that centrifugation can mitigate the inhibitory effect of apolipoproteins on αSyn seeding.

To validate our findings, we mixed PD patient serum with PBS (0.02% NaN₃, pH 7.3) and 1 × Protease Inhibitor Cocktail, followed by centrifugation at 21,000 × *g* for 30 min at room temperature. The lower supernatant was collected, and streamlined SAA was performed by adding 5 µL of each sample to a reaction mixture containing αSyn monomer in a total volume of 50 µL per well. LDL and HDL were reduced by 50% and 30%, respectively, and SAA analysis detected αSyn seeds in 30% (3/10) of PD serum samples (Fig. S3). A second centrifugation step further reduced LDL and HDL by 80% and 50%, respectively, and αSyn seed detection rate was increased to 80% (8/10) of PD samples (Fig. S3). Transmission electron microscopy confirmed reduction of lipid-related components, and Western blot showed reduction of ApoA1 and ApoE in the bottom supernatant after dilution-centrifugation compared to the untreated serum (Fig. S4a–c). Importantly, none of the HC serum (*n* = 5) met the positivity criteria. These results show that dilution-centrifugation reduces inhibitors of SAA, improves assay sensitivity and provides a lipo-free protocol for better detection.

To assess the sensitivity and specificity of lipo-free SAA, we performed a blinded lipo-free SAA of an additional panel of serum samples (Fig. 1d). A total of 102 PD serum samples and 185 HC samples were tested, with demographic and clinical information provided in Table S2. The lipo-free SAA demonstrated 80.39% sensitivity and 92.43% specificity. ThT kinetics revealed a significantly higher PAR in PD samples compared to controls (Fig. 1e, f; *P* < 0.0001). Receiver operating characteristic analysis demonstrated an accuracy of 0.8854 (AUC 95% CI 0.8375–0.9332, *P* < 0.0001) for diagnosing PD (Fig. 1g). These results highlight that αSyn seed detection in serum using this protocol is both highly sensitive and specific for distinguishing PD from HC.

A recent study by the Okuzumi group has shown that blood could be a useful diagnostic tool for synucleinopathies [3]. They used an IP-based SAA (IP/SAA) assay and found that αSyn seeds are present in the serum of PD patients. This supports the idea that serum can be used to detect αSyn seeds and help diagnose synucleinopathies. Additionally, studies have found that brain homogenates and CSF can inhibit amyloid formation, with lipid-related substances identified as key inhibitors [5, 7]. Our findings demonstrate that apolipoproteins, particularly ApoE, suppress αSyn-seeded amyloid formation by interacting with αSyn and slowing its aggregation, a process reversible by centrifugation. This method offers simplicity, lower cost, and reduced processing time, making it a practical alternative. However, the current study has some limitations, including a small sample size, focusing on late-stage PD, and lower rate of αSyn seed detection which may be due to residual inhibitors or co-pathologies [8]. Future research should involve larger cohorts and optimized protocols for improved early-stage diagnosis and deeper insights into αSyn in PD.

## Supplementary Information

Additional file 1. Materials and methods.

Additional file 2. **Figure S1.** Different serum fractions affect αSyn aggregation differently. **Figure S2**. Lipoproteins slow down αSyn-seeded amyloid formation. **Figure S3**. Successful seeding in serum in αSyn SAA after removing lipoproteins. **Figure S4**. Characterization of serum samples following sequential dilution and centrifugation.

Additional file 3. **Table S1**. Identification of proteins in the >100 kDa fraction of human serum by liquid chromatography-mass spectrometry.

Additional file 4. **Table S2**. Demographics of study participants and results of αSyn lipo-free SAA in serum samples.

Additional file 5. Gels and Blots for Fig. S4.

## Data Availability

Any additional information required to reanalyze the data reported in this report is available from the lead contact upon request. All materials used in this study will be made available subject to a materials transfer agreement.

## Abbreviations

αSyn: Alpha-synuclein
SAA: Seed amplification assay
PD: Parkinson's disease
hPFFs: Human αSyn pre-form fibrils
ThT: Thioflavin T
PAR: Protein aggregation rate
HDL: High-density lipoprotein
LDL: Low-density lipoprotei
IP: Immunoprecipitation
