# Supplementary material for: Enhanced serum-based seed amplification assay for detecting propagative α-synuclein seeds in Parkinson’s disease
Source: Transl Neurodegener. 2025 May 22;14:24. doi: 10.1186/s40035-025-00488-3 (PMC12096493; doi:10.1186/s40035-025-00488-3)
Supplement: Supplementary file 1 — Additional file 1. Materials and methods. [file 40035_2025_488_MOESM1_ESM.docx]

**Supplementary materials**

**Materials and methods**

**Recombinant human/mouse αSyn Monomer Purification.** The human/mouse αSyn was purified following a previously described protocol with slight modifications^1^. The BL21 (DE3) E. coli was transformed with a plasmid encoding full-length SNCA/Snca and the bacteria was grown overnight at 230 rpm until the optical density at 600 nm reached 0.8. Protein expression was then induced with 1.0 mM isopropyl β-D-thiogalactoside (IPTG) for 4 hours. After induction, the cells were harvested by centrifugation at 8000× g for 10 minutes at 4 °C. The resulting bacterial pellets were resuspended in a high salt buffer (10 mM Tris-HCl, pH 7.5, 500 mM NaCl, 1 mM EDTA, 1 mM PMSF, and protease inhibitors) and sonicated on ice. The lysate was then boiled for 10 minutes and subjected to ultracentrifugation at 20,000× g for 30 minutes at 4 °C. The supernatant was treated with 45% saturated ammonium sulfate to precipitate proteins, which were then resuspended in 10 mM Tris-HCl (pH 7.5) and 50 mM NaCl. The pH of the solution was adjusted to 3.0 using HCl to remove unwanted proteins by centrifugation, followed by readjusting the pH to 7.5 with NaOH. The protein solution was further purified using a HiTrap column and dialyzed against 10 mM Tris-HCl (pH 7.5) with 50 mM NaCl. Finally, the sample was filtered through a 100-kDa Amicon Ultra filter to ensure the removal of any high molecular weight contaminants. In this experiment, we used the same batch of monomers for SAA.

**Generation of αSyn PFFs from recombinant αSyn.** Purified human αSyn monomer was used to generate αSyn hPFFs according to the previous protocol^2^. Briefly, the human αSyn monomer was agitated at 1000 rpm for seven consecutive days (Eppendorf, Hamburg, Germany) to generate αSyn hPFFs. The resulting hPFFs were then sonicated for 10 minutes before use. In this experiment, we used the same batch of hPFFs for further experiment.

**Depletion and isolation of serum ApoA1, ApoE, and αSyn by immunoprecipitation.** To deplete ApoA1 and ApoE from a serum sample, immunoprecipitation (IP) was carried out as follows: For ApoA1 depletion, 50 μL of Protein A/G Magnetic Beads (Thermo Fisher, CA, USA) was mixed with 100 μg of anti-Apo A1 antibody(Thermo Fisher, CA, USA) diluted in 100 µl of IP lysis buffer (1% BSA, 150 mM NaCl, 1% Triton X, 50 mM Tris-HCl, pH 7.0) in a 2-mL tube and incubated at RT for 2 h with constant rotation. The mixture was centrifuged at 1,000×g for 5 minutes, the supernatant was discarded, and the beads were washed three times with TBST. For ApoE depletion, a similar process was followed using 50 μL of beads and 80 μg of anti-ApoE antibody (Millipore, Darmstadt, Germany). 200 μL serum sample (containing 1 mg of protein per milliliter) was diluted 4 times and added to the beads. After overnight incubation at 4°C with constant rotation, the mixture was centrifuged again, and the supernatant was collected. The antibody-antigen complexes bound to the beads were then eluted by incubating the beads with 100 µl of 0.2% glycine (pH 2.6) for 10 minutes with agitation. The eluates were subsequently neutralized by adjusting the pH to 7.5. A control sample was prepared by treating beads without antibodies in the same manner.

For the IP of αSyn, 100 µl of IP lysis buffer was prepared by adding 2 µg of the MJFR-1 anti-αSyn antibody (Abcam, MA, USA) and 30 µl of protein A/G magnetic beads (Thermo Fisher Scientific, MA, USA). This mixture was incubated for 2 hours at RT, followed by centrifugation to remove unbound components. Next, 200 µl of serum (with a protein concentration of 1 mg/ml) was diluted 1:2 with lysis buffer and added to the antibody-bead complex. The mixture was rotated at 4 °C overnight to allow antigen-antibody binding. The antibody-antigen complexes bound to the beads were then eluted by incubating the beads with 30 µl of 0.2% glycine (pH 2.6) for 10 minutes with agitation. The eluates were subsequently neutralized by adjusting the pH to 7.5.

**ELISA for Detecting ApoE and αSyn.** A ELISA was performed by coating plates with either goat anti-ApoE antibody (Millipore, Darmstadt, Germany) or rabbit anti-αSyn antibody (Santa Cruz, California, USA), with rabbit IgG as controls, followed by the addition of human serum, incubation with detection antibodies (anti-ApoE or anti-αSyn), with uncoated wells and IgG controls ensuring specificity.

**Measure HDL and LDL concentrations.** The concentrations of HDL and LDL were measured according to the manufacturer’s instructions. Standard solutions and samples were prepared following the protocols provided with the HDL (ELK Biotechnology, Wuhan, China) and LDL/VDLD (Solarbio, Beijing, China) kits. Substrate was added to initiate the color reaction, and the concentrations of HDL and LDL were calculated based on the generated standard curve.

**Serum Fractionation procedure.** An aliquot of 0.5 ml of serum was resuspended in 14.5 ml of PBS to obtain 15 ml of diluted serum. After filtering through a 0.22-micron filter, the serum was subjected to a series of filtrations using Amicon® Ultra4 molecular weight cut-off (MWCO) filters. The process of human serum fractionation is depicted schematically in Figure S1a.

**Serum Sample.** A total of 287 samples were collected from the Department of Neurology at the First Affiliated Hospital of Guangzhou Medical University between 2023 and 2024. These samples included 102 subjects who received a clinical diagnosis of probable PD based on the international diagnostic criteria and 185 age-matched healthy controls (HC)^3^. Diagnostic criteria adhered to established norms, with control subjects exhibiting no history of parkinsonism or signs of synucleinopathy. In our experimental setup, each participant underwent a comprehensive clinical research assessment, which included evaluations of cognitive and motor functions. Cognitive function was assessed using the Mini-Mental State Exam (MMSE), while motor function was evaluated through a structured physical neurological examination. This examination placed particular emphasis on motor assessments using the Hoehn and Yahr scale and the Unified Parkinson’s Disease Rating Scale (UPDRS) Part III motor assessment. All participants were enrolled in a research protocol that provided for yearly follow-up assessments, ensuring that each participant received at least one follow-up assessment after their initial visit. Neuroimaging results, such as MRI and occasionally FDG PET scans or DaTscan, were reviewed when available. Diagnoses for each group were initially determined by movement disorder specialists at the respective sites and subsequently verified by a central consensus committee. Notably, αSyn SAA results were not accessible to investigators or the consensus committee during the diagnosis process and were therefore not considered in the classification of participants.

Blood samples were centrifuged at 3,000g for 15 minutes at 4 °C, aliquoted, and stored at −80 °C until analysis. The sample collection methodology was approved by the Ethics Committee of the First Affiliated Hospital of Guangzhou Medical University (ES-2024-216-01). All study participants provided written informed consent.

**Lipo-free Serum Preparation.**

In a laboratory setting, 200 µL of serum was mixed with an equal volume of PBS containing 0.02% NaN₃ (pH 7.3, buffer solution) and 1× Protease Inhibitor Cocktail (CWBIO, China). The mixture was then centrifuged at 14,000g for 30 minutes at RT using Axygen® 1.5 mL Snaplock Microcentrifuge Tubes (Corning) in an Eppendorf 5430R microcentrifuge equipped with the FA-45-30-11 fixed-angle rotor (45° angle relative to the vertical axis). After centrifugation, a sterile pipette tip was carefully inserted into the tube without disturbing the supernatant, and 100 µL of the lower liquid phase was transferred into a new sterile tube. This was then mixed with another 100 µL of buffer solution, bringing the total volume to 200 µL, followed by a second round of centrifugation under the same conditions. After the second centrifugation, another 100 µL of the lower supernatant was carefully extracted using a sterile pipette tip, ensuring minimal disruption, as shown in SFig.4A. The collected samples were aliquoted for SAA analysis.

**Streamlined SAA.** The streamlined SAA was built according to the previous protocol^1,4^. A 5 µL aliquot of each sample was added to a reaction mixture containing 0.1 mg/mL mouse αSyn monomer, 10% ammonium sulfate, 40 mM Thioflavin T (ThT), 50 mM NaCl, and 10 mM Tris (pH 7.5), resulting in a total volume of 50 µL per well in a black 384-well plate with a clear bottom (Thermo Fisher, CA, USA). Each sample was tested in triplicate. The plate was sealed with sealing film (Thermo Fisher, CA, USA) and incubated at 50°C in a SpectraMax iD5 plate reader (Molecular Devices, CA, USA) with intermittent shaking at 600 rpm (1-minute shaking followed by a 14-minute rest period) throughout the assay. ThT fluorescence was measured using 450 ± 10 nm excitation and 490 ± 10 nm emission. A sample was considered positive if at least two out of three replicates exceeded the threshold of background fluorescence plus 10 standard deviations, corresponding to an approximate 11.6% cut-off threshold within 24 h. This algorithm was defined prior to the analysis.

**Transmission Electron Microscopy.** For transmission electron microscopy (TEM) of serum samples, a 10 μL volume was loaded onto freshly glow-discharged 400-mesh carbon-coated copper grids (Electron Microscopy Sciences, PA, USA) and incubated for two minutes. The grid was then quickly rinsed with three drops of 50 mM Tris-HCl (pH 7.5) and floated on two drops of 0.75% uranium formate (Solarbio, Beijing, China) for staining. Imaging was performed using a CM 120 transmission electron microscope (PHILIPS, Eindhoven, The Netherlands) at an acceleration voltage of 80-120 kV.

**Western Blot Analysis**. Purified protein samples (20 µg/well) were mixed with SDS loading buffer (Biyotime, Beijing, China) and boiled at 95°C for five minutes before separation on a 15% SDS-polyacrylamide gel. After electrophoresis, proteins were transferred to polyvinylidene fluoride (PVDF) membranes (Millipore, Darmstadt, Germany). The membranes were blocked in 5% BSA diluted in Tris-buffered saline with 1% Tween-20 (TBST) for one hour at room temperature. They were then incubated overnight with primary antibodies, including anti-ApoE antibody (Millipore, Darmstadt, Germany) and anti-ApoA1 antibody (Thermo Fisher, CA, USA). After washing with TBST, the membranes were incubated for one hour with horseradish peroxidase-conjugated secondary antibodies (Bio-Rad, Hercules, CA) diluted 1:5000 in blocking buffer. Immunoblots were developed using IMMOBILON-enhanced chemiluminescence (Millipore, Darmstadt, Germany), and contrast adjustments were uniformly applied in Adobe Photoshop to enhance band visibility.

**LC-MS/MS Analysis.** The LC-MS/MS analysis of tryptic peptides was performed using a quadrupole Orbitrap mass spectrometer (ORBITRAP ECLIPSE, Thermo Fisher Scientific, USA) coupled to an EASY nLC 1200 ultra-high pressure system (Thermo Fisher Scientific) via a nano-electrospray ion source. A total of 1 µg of peptides was loaded onto a 25 cm column with an inner diameter of 100 µm, packed with ReproSil-Pur C18-AQ 1.5 µm silica beads (QL-HPLC-100*15). The peptides were separated using a gradient elution as follows: 6% to 12% solvent B over 15 minutes, then 12% to 30% solvent B over 48 minutes, followed by 30% to 40% solvent B over 10 minutes. The gradient was then stepped up to 95% solvent B in 1 minute, maintained at 95% solvent B for 10 minutes, and finally reduced to 6% solvent B in 1 minute. The flow rate was maintained at 300 nl/min. Solvent A consisted of 0.1% formic acid in water, while solvent B was composed of 80% acetonitrile and 0.1% formic acid in water.

Mass spectra were acquired using the ORBITRAP ECLIPSE Mass Spectrometer (Thermo Fisher Scientific). MS1 spectra were collected at a resolution of 120,000, with a scan range from 350 to 1500 m/z. The normalized AGC target was set to 4E5, and the maximum injection time was 50 ms. MS2 spectra were acquired with a normalized AGC target of 5E4 or a maximum injection time of 22 ms. The peptides were fragmented using 33 normalized HCD collision energy, and the resulting spectra were acquired at a resolution of 15,000, with a first mass of 200 m/z.

**Statistical analysis.** All statistical analyses were performed using GraphPad Prism Software (GraphPad Software Inc., CA, USA). For comparisons between the two groups, the two-tailed independent Student's t-test was employed. For analyses involving three or more groups, a one-way analysis of variance (ANOVA) was conducted, followed by Tukey's post-hoc test. Unless otherwise specified, data are expressed as mean values accompanied by their standard deviation (SD). A P value of less than 0.05 was considered statistically significant, indicating meaningful differences between groups. This approach ensured robust statistical evaluation and interpretation of the experimental results.

1. Mao H, Kuang Y, Feng D, et al. Ultrasensitive detection of aggregated α-synuclein using quiescent seed amplification assay for the diagnosis of Parkinson’s disease. *Translational Neurodegeneration*. 2024/07/24 2024;13(1):35. doi:10.1186/s40035-024-00426-9

2. Volpicelli-Daley LA, Luk KC, Lee VM. Addition of exogenous alpha-synuclein preformed fibrils to primary neuronal cultures to seed recruitment of endogenous alpha-synuclein to Lewy body and Lewy neurite-like aggregates. *Nat Protoc*. Sep 2014;9(9):2135-46. doi:10.1038/nprot.2014.143

3. Postuma RB, Berg D, Stern M, et al. MDS clinical diagnostic criteria for Parkinson's disease. *Mov Disord*. Oct 2015;30(12):1591-601. doi:10.1002/mds.26424

4. Kuang Y, Mao H, Gan T, et al. A skin-specific alpha-Synuclein seeding amplification assay for diagnosing Parkinson's disease. *NPJ Parkinsons Dis*. Jul 4 2024;10(1):129. doi:10.1038/s41531-024-00738-7
