# Supplementary material for: Enhanced serum-based seed amplification assay for detecting propagative α-synuclein seeds in Parkinson’s disease
Source: Transl Neurodegener. 2025 May 22;14:24. doi: 10.1186/s40035-025-00488-3 (PMC12096493; doi:10.1186/s40035-025-00488-3)
Supplement: Supplementary file 2 — Additional file 2. Figure S1. Different serum fractions affect αSyn aggregation differently. Figure S2. Lipoproteins slow down αSyn-seeded amyloid formation. Figure S3. Successful seeding in serum in αSyn SAA after removing lipoproteins. Figure S4. Characterization of serum samples following sequential dilution and centrifugation. [file 40035_2025_488_MOESM2_ESM.docx]

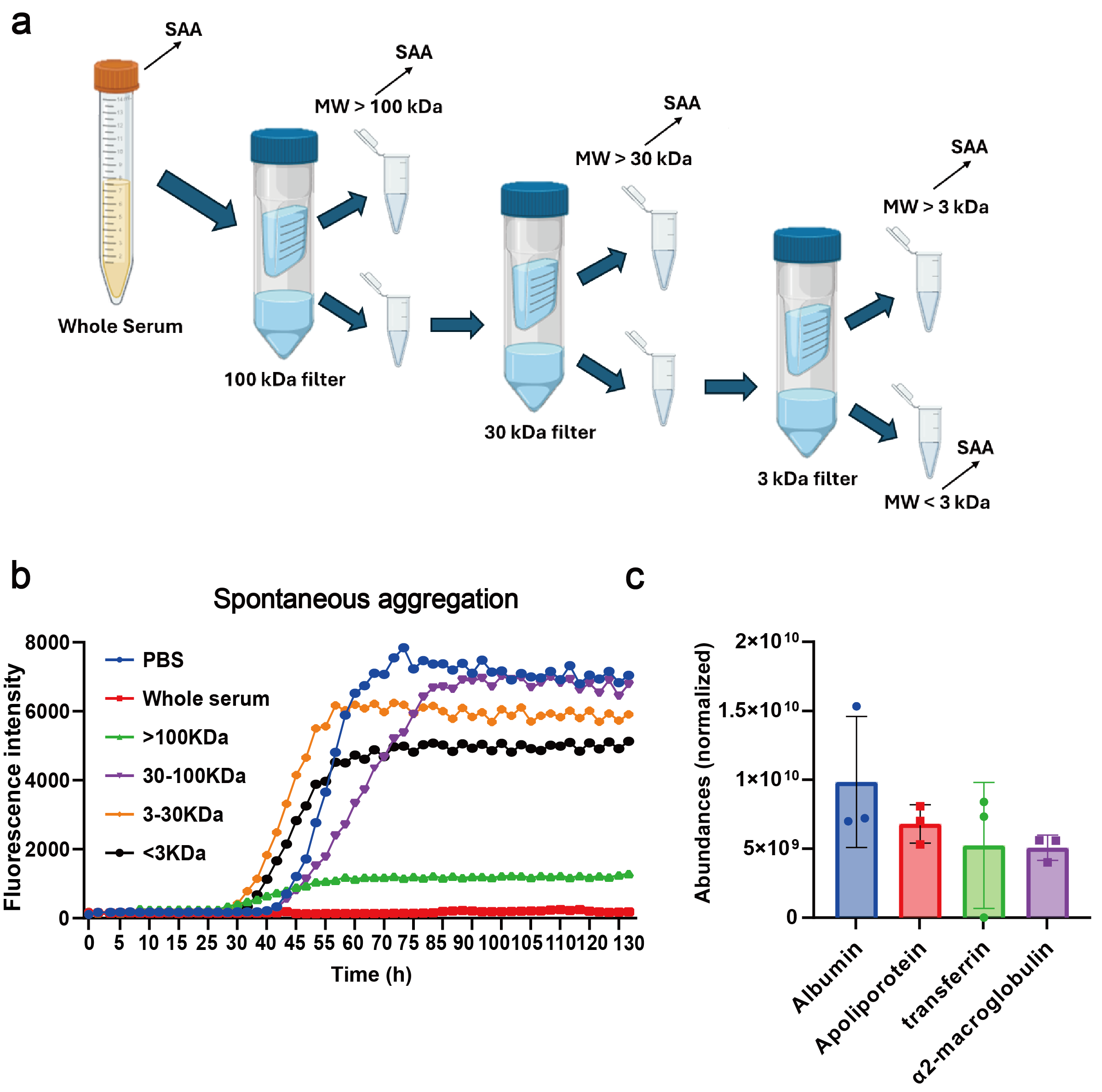


**Figure S1. Different serum fractions affect αSyn aggregation differently. a** The image outlines a serum fractionation procedure starting with an aliquot of 0.5 mL of serum mixed with 14.5 mL of PBS. The process involves collecting four aliquots containing compounds of different molecular weights through filtration using centrifugal filters. The flow-through of each filtered fraction is passed to a filter with a smaller cut-off. **b** Representative image of ThT protein aggregation in the presence of different serum fractions and whole serum to evaluate effects of serum on αSyn spontaneous aggregation. **c** Relative concentrations of the most abundant protein constituents over 100 kDa measured by liquid chromatography-mass spectrometry. SAA: Seed amplification assay, **ThT**: Thioflavin T


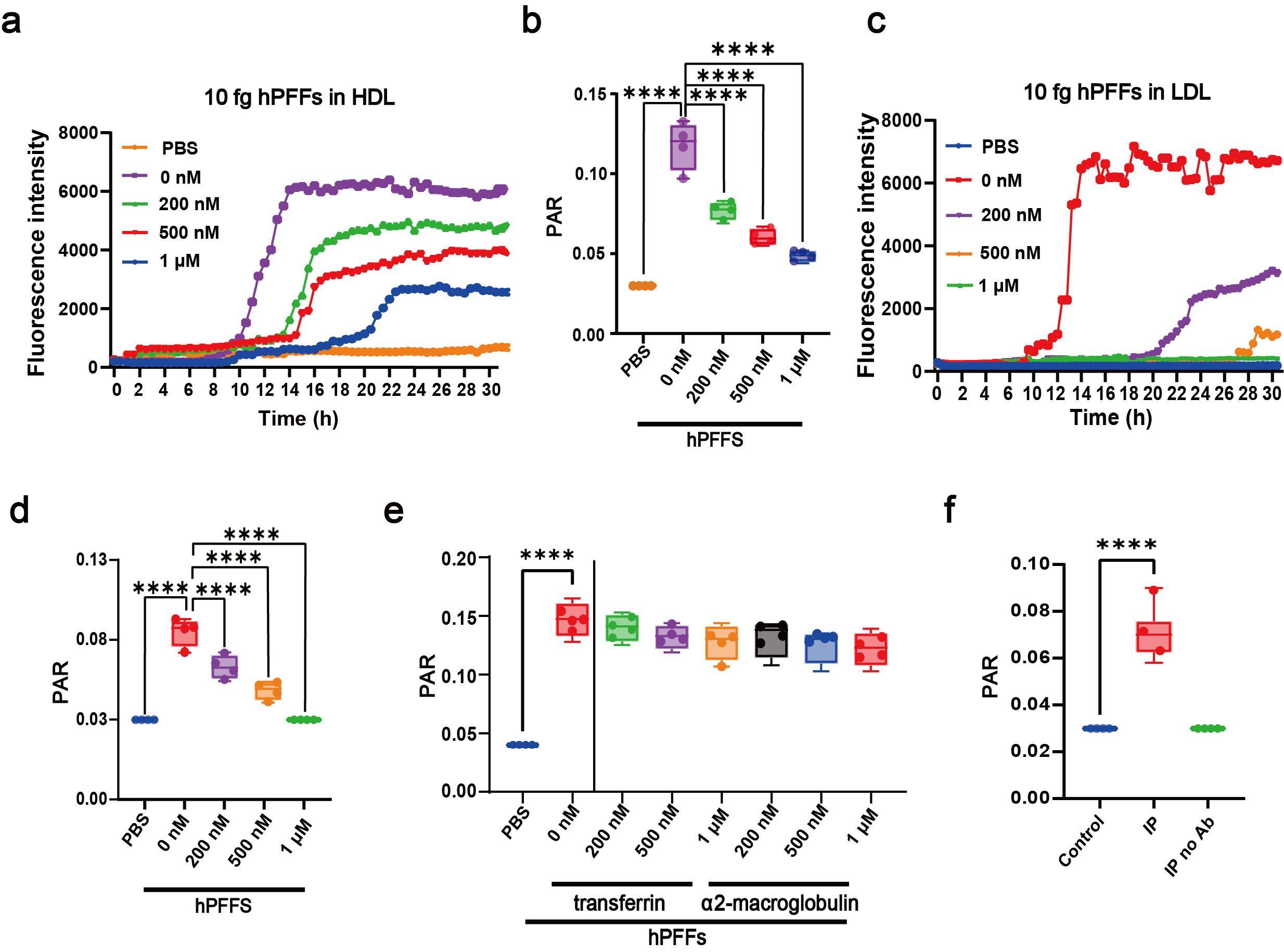


**Figure S2: Lipoproteins slow down αSyn-seeded amyloid formation. a** Representative kinetic curves of SAA with different concentrations of HDL (0, 200 nmol/L, 500 nmol/L, and 1 μmol/L) when 10 fg of hPFFs was added in each well. **b** PAR of the hPFFs by SAA assay with addition of HDL (0, 200 nmol/L, 500 nmol/L, and 1 μmol/L) in the presence of 10 fg of hPFFs (n=4). **c** Representative kinetic curves of SAA with different concentrations of LDL (0, 200 nmol/L, 500 nmol/L, and 1 μmol/L) when 10 fg of hPFFs was added in each well. **d** PAR of the hPFFs by SAA assay with addition of LDL (0, 200 nmol/L, 500 nmol/L, and 1 μmol/L) in the presence of 10 fg of hPFFs (n=4). **e** Comparison of the PAR in the presence of different concentrations of purified transferrin and α2-macroglobulin (0, 200 nmol/L, 500 nmol/L, and 1 μmol/L) when 10 fg of hPFFs were present in the each well (n=4). **f** PAR levels in serum samples after immunoprecipitation of ApoA1 and ApoE, followed by incubation with 10 fg of hPFFs. Untreated serum and IP without an antibody served as control groups (n=3). Data are presented as mean ± SD of all samples analyzed in each group. One-way ANOVA followed by Tukey's post-hoc test was used. **P* < 0.05, ***P* < 0.01, ****P* < 0.001, and *****P* < 0.0001. SAA: seed amplification assay, hPFFs: human-derived preformed fibrils, HDL: high-density lipoprotein, LDL: low-density lipoprotein, ApoA1: apolipoprotein A1, ApoE: apolipoprotein E.


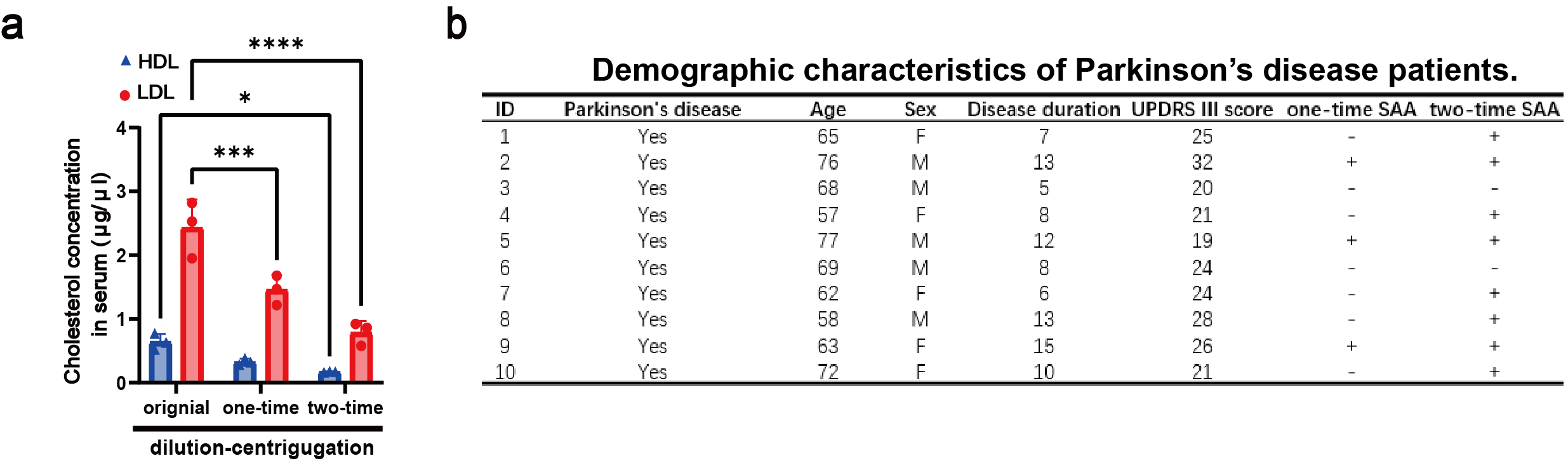


**Figure S3.** **Successful seeding in serum in αSyn SAA after removing lipoproteins. a** HDL and LDL concentrations after one or two rounds of dilution-centrifugation applied to PD serum (n=3). **b** Demongraphics of HC and PD patients and SAA results after one or two times of dilution-centrifugation. One-way ANOVA followed by Tukey's post-hoc test. Data are shown as mean ± SD. *P < 0.05, **P < 0.01, ***P < 0.001, and ****P < 0.0001. SAA: Seed Amplification Assay, HDL: High-Density Lipoprotein, LDL: Low-Density Lipoprotein


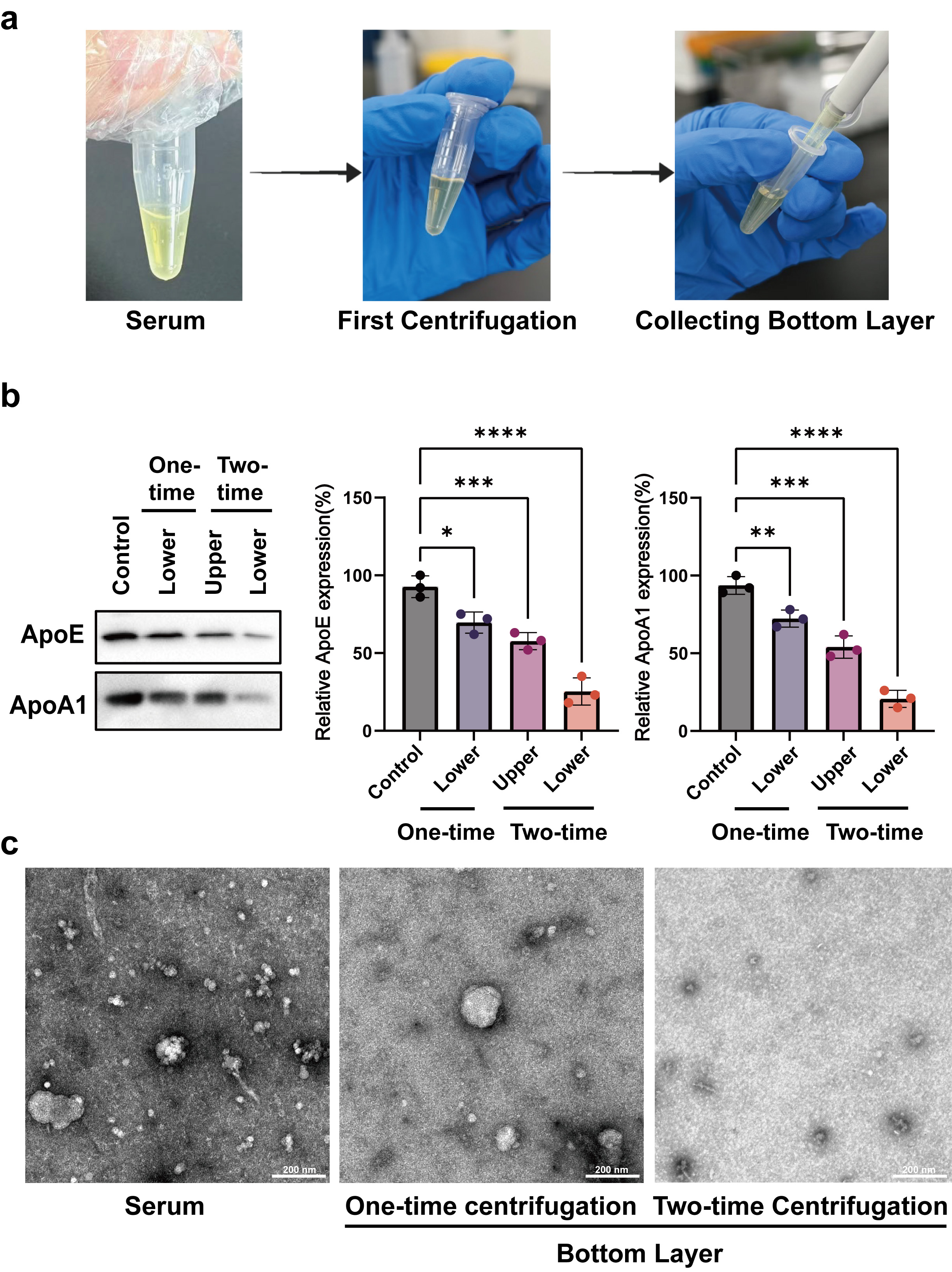


**Figure S4. Characterization of serum samples following sequential dilution and centrifugation. a** Representative photos of human serum before and after the first dilution-centrifugation, and collection of the bottom layer after centrifugation. **b** Western blots of ApoA1 and ApoE following the first and the second dilution-centrifugation, with untreated serum as the control (n=3). The right panels present quantification. **c** Transmission electron microscopy images illustrating the morphological changes in the sample after the first and the second dilution-centrifugation, compared to untreated serum. Scale bar, 200 nm. One-way ANOVA followed by Tukey's post-hoc test. Data are shown as mean ± SD. *P < 0.05, **P < 0.01, ***P < 0.001, and ****P < 0.0001. ApoA1, Apolipoprotein A1; ApoE, Apolipoprotein E
