# Supplementary material for: Enhanced serum-based seed amplification assay for detecting propagative α-synuclein seeds in Parkinson’s disease
Source: Transl Neurodegener. 2025 May 22;14:24. doi: 10.1186/s40035-025-00488-3 (PMC12096493; doi:10.1186/s40035-025-00488-3)
Supplement: Supplementary file 4 — Additional file 4. Table S2. Demographics of study participants and results of αSyn lipo-free SAA in serum samples. [file 40035_2025_488_MOESM4_ESM.docx]

**Table S2 Demographics of study participants and results of αSyn lipo-free SAA in serum samples**

|  | **PD (*n*=102)** | **Non-synucleinopathy Controls (*n*=185)** |
| --- | --- | --- |
| Age (years), mean (SD) | 66.4 (9.2) | 65.2 (10.8) |
| Sex, male (female) | 54 (48) | 95 (90) |
| Duration of disease (years) | 7.9 (4.32) | NA |
| Hoehn and Yahr scale | 2.9 (0.85) | NA |
| UPDRS III | 35.2 (18.25) | NA |
| Lipo-free SAA positive (*n*) | 82 | 14 |
| Lipo-free SAA negative (*n*) | 20 | 171 |
| Sensitivity | 80.39% |  |
| Specificity | 92.43% |  |

PD, Parkinson’s disease; NA, not applicable SAA; Seed amplification assay
