# Supplementary figures and images for: Enhanced serum-based seed amplification assay for detecting propagative α-synuclein seeds in Parkinson’s disease
Source: Transl Neurodegener. 2025 May 22;14:24. doi: 10.1186/s40035-025-00488-3 (PMC12096493; doi:10.1186/s40035-025-00488-3)

**Fig. S4**

**ApoA1**

**
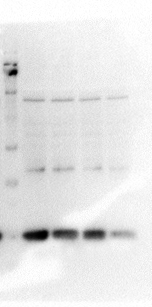
**

**ApoE**

**
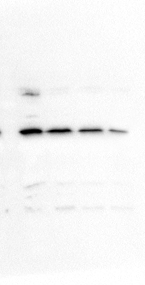
**

Supplement: Supplementary file 5 — Additional file 5. Gels and Blots for Fig. S4. [file 40035_2025_488_MOESM5_ESM.docx]
